# Behind the scenes: a qualitative study on threats and violence in emergency medical services

**DOI:** 10.1186/s12873-024-01090-y

**Published:** 2024-09-26

**Authors:** Isabelle Stjerna Doohan, Måns Davidsson, Martin Danielsson, Jonas Aléx

**Affiliations:** 1 Unit of Police Work, Umeå University, Umeå, Sweden; 2 Department of Nursing, Umeå University, Umeå, SE-901 87 Sweden

**Keywords:** Nurses, Violence, Threat, Prehospital, Ambulance, Ambulance clinicians, Police, Collaboration

## Abstract

The increasing prevalence of threats and violence against ambulance clinicians is a critical issue that has not been adequately studied. These incidents pose significant challenges to the provision of prehospital emergency care, affecting both the safety and well-being of the clinicians involved. This study aimed to explore the experiences of Swedish ambulance clinicians when encountering threats and violence during their work. A qualitative approach was used, involving semi-structured interviews with 11 ambulance clinicians from various regions of Sweden. The participants were selected to ensure diversity in gender, age, and educational background. The data were collected over three weeks in 2021 and analyzed using qualitative content analysis. The analysis revealed three key categories related to the challenges faced by ambulance clinicians: Police cooperation challenges, Strategies for a safe care environment, and Impact during and relief after stressful events. These categories highlight the complexities of managing threats and violence in the field. This study sheds light on the multifaceted challenges that ambulance clinicians face due to threats and violence. It underscores the urgent need for comprehensive training, effective communication, and clear role allocation in complex situations. Furthermore, it emphasizes the importance of organized support systems to help clinicians cope with the aftermath of stressful events.

**Supplementary Information:**

The online version contains supplementary material available at 10.1186/s12873-024-01090-y.

## Background

Ambulance care refers to the care and treatment provided by ambulance clinicians in the emergency medical services (EMS). This care takes place outside formal health care institutions, often in the patient’s home. Healthcare professionals working in the EMS differ worldwide. In Sweden, ambulances are staffed by at least one Registered Nurse (RN) due to regulations on the administration of drugs. The ambulances are staffed with RNs who hold either a bachelor’s degree or an RN with an additional year of training in emergency care at an advanced level, including a one-year master’s degree. The team in the ambulance consists of two RNs, or one RN and one emergency medical technician, who possess basic life support competencies. In this article ambulance clinicians refer to all personnel working in EMS. Ambulance clinicians face many different stressors in their work, and two of them are threats and violence [1]. Threats and violence against ambulance clinicians working in EMS constitute a growing problem that has not received adequate attention [2].

In the US, ambulance clinicians face an alarming rate of occupational violence-related injuries, approximately six-times higher compared to all US workers [3]. International studies indicate that a minimum of 65% of ambulance clinicians experience workplace violence annually [4–6]. For instance, in a study focused on ambulance clinicians in Spain, 34.5% of participants reported exposure to physical violence from patients and their relatives, while over 75% faced verbal abuse from the same groups [7]. Occupational violence within the EMS context is a concern, with most ambulance clinicians reporting some form of abuse, intimidation, or even physical or sexual assault during their careers [8]. A recent report on threats and violence against ambulance clinicians, conducted by the Swedish Work Environment Authority, reveals an increase in prevalence from 2018 to 2022 [9].

EMS assignments often present complex scenarios that challenge ambulance clinicians’ decision-making skills [10]. Threats and violence against ambulance clinicians constitute a serious workplace issue with repercussions for both the individual and society at large. A deterioration in patient care in EMS settings due to threats and violence has been found as early as a decade ago [11]. Additionally, ambulance clinicians are encountering an increasing number of patients suffering from mental illness [12]. Working under the constant risk of violence can detrimentally impact the quality of the care provided [13]. This may manifest in ambulance clinicians treating patients with heightened suspicion and vigilance [11] or expressing a reluctance to remain in the workplace [14].

In a study investigating Swedish trauma hospital nurses’ encounters with workplace violence and threats, it was noted that experiencing threatening situations, including insults, threats, and intimidating behaviour, can lead to feelings of insecurity, anxiety, and a persistent fear of encountering threats or violence beyond the workplace. The effects are not limited to professional life; personal lives are also affected, as exposed nurses often seek care for the resulting emotional distress [14].

Despite the prevalence of threats and violence in emergency care, this issue remains inadequately addressed [2, 15]. The prehospital literature acknowledges the severity of this issue and hints at mitigation strategies, such as hazard flagging, training programmes and training simulations, but further research is needed to explore the mechanisms and effectiveness of these interventions [8, 16, 17]. There is a need to deepen our understanding of ambulance clinicians’ experiences of working under such conditions. By shedding light on these experiences, the study aims to serve as a foundation for refining routines, training, and preparedness for handling threats and violence. Furthermore, it can yield insights into these situations and the experiences of ambulance clinicians, ultimately providing the knowledge needed to mitigate stressors for personnel, ensuring their ability to work safely and deliver high-quality care in demanding circumstances.

### Aim

The aim of this study was to explore ambulance clinicians’ encounters with threats and violence while providing prehospital emergency care.

## Methods

### Design

This is a qualitative interview study with Swedish nurses in EMS. Qualitative methods were chosen to comprehensively explore and understand their experiences of working in environments with threats and violence. The study followed an inductive approach, commencing the analysis from the empirical data, examining it without preconceived notions, and describing it as accurately as possible [18]. To ensure the study’s rigor the COREQ-checklist developed from Tong et al. was used [19]. (See Supplementary material).

### Settings and participants

The interviews were conducted in Sweden with 11 participating ambulance clinicians from different parts of the country. Among the participants, nine were male and two were female, with ages ranging from 28 to 54 years. Their educational backgrounds varied: five were registered nurses, while six held a nursing degree with at least one year of specialized education in emergency care. Additionally, five participants had supplemental military experience as nurses during military missions abroad. They collectively possessed experience working in both urban and sparsely populated areas. The inclusion criteria mandated that participants must have worked as ambulance clinicians for at least one year and must have experienced at least one situation involving threats and violence during a job assignment in an EMS setting (in either a civilian or military context). In both the results and the discussion, no distinction was made between participants based on their civilian or military backgrounds.

Initially, a convenience sample method was used to efficiently reach participants who met the inclusion criteria. This involved direct requests to known ambulance nurses, which yielded seven participants. Additionally, a request for participation was posted on a social media forum for veterans in Sweden, resulting in two more participants. Subsequently, a snowball selection method was employed to contact three additional individuals who met the inclusion criteria.

### Data collection

The participants were interviewed via a digital platform, and all interviews were video recorded. The interviews were conducted by the authors MD and MD, who were Master students in ambulance care. Prior to the interviews, verbal consent was obtained from the participants. An interview guide was developed for this study specifically (see Supplementary material) and it was developed by all authors. A pilot interview was conducted, and the interview guide was revised afterwards. After initial demographic inquiries, the participants were prompted with an open-ended question: “Can you describe a complex situation where you worked with prehospital emergency care under threats and violence?” Depending on what the participants said, follow-up questions were asked, such as: “How did it feel? Which experiences did you take with you?” Questions were also asked about how the collaboration worked. The interviews were conducted during three weeks in October 2021. The length of the interviews varied between 28 and 77 min. The interviews were transcribed by MD and MD.

### Data analysis

The data analysis followed a triangulation process to ensure rigor and trustworthiness of the study. The empirical data was analyzed using qualitative content analysis [20]. In the analysis process, the transcribed text was repeatedly reviewed, searching for patterns, similarities, and differences, with notes made in the margin to initiate data structuring. Subsequently, meaning units were selected that corresponded to the aim of the study. These units were condensed and manually coded to describe the content while retaining the core message. Finally, the codes were grouped into larger sets to form subcategories and categories. These were clarified in meaning units whose content was then interpreted and processed together to form nine subcategories and three categories. To minimize the risk of misinterpretation based on the authors’ preunderstanding, the interviews were then reviewed. The processing of meaning units was carried out using a table system. In line with a triangulation process, the analysis continued between the authors throughout the entire writing process, moving back and forth between the transcribed data and the formulated themes. The analysis and presentation of results was also reviewed by two senior researchers from the research network Emergency Care, situated at Umeå University.

Authors MD and MD conducted the data analysis, supported by authors JA and ISD. The analysis was initially performed in Swedish and subsequently translated into English. Three of the authors (JA, MD, MD) have experience in military and/or civilian EMS and have interpreted the codes collectively with ISD through discussion.

## Results

The 11 participants had diverse experiences, including caring for patients with gunshot or knife wounds in situations with active threats on site, spanning locations like Afghanistan, Mali, and Sweden. Participants also described situations where patients had been violent and posed threats, including encounters with agitated pit bulls, knives, and bottles thrown as they fled, sudden violence during transport, wrestling with patients holding razor blades, and verbal threats. Many of the participants reported feeling genuinely scared.

The results consist of three categories followed by nine subcategories (Table 1). The categories are Police cooperation challenges, Strategies for a safe care environment, and Impact during and relief after stressful events.

**Table 1:** Overview of categories and subcategories

Category	Subcategory
Police cooperation challenges	• Threats are perceived differently • Being without police support generates frustration • Varying outcomes when cooperating with the police
Strategies for a safe care room	• Mental preparation and prioritizing personal safety • Impact of Previous Experience on Protective Traits • Role Allocation and Effective Communication Enhance Collaboration
Impact during and relief after stressful events	• Desire for specific and recurrent training • Significance of collegial and social support • Need for organized support

### Police cooperation challenges

In the EMS setting, various skills are necessary as threats and violent situations are dynamic events with continuous potential to escalate. Identifying threats can be challenging as they can arise unexpectedly. Participants perceived cooperation between ambulance clinicians and the police as crucial for performing emergency medical care in threatening and violent situations. Collaboration has factors that either facilitate or complicate, such as joint training sessions, personal knowledge, and the patient’s attitude toward the police.

#### Threats are perceived differently

Perception of threatening situations varies based on individuals and their past experiences. What seems dangerous to one person might feel safe to another, such as dealing with an aggressive dog or a violent patient. Exposure to threats and violence can evoke different reactions, leading to fear, anxiety, or altered decision-making during work. The dynamic nature of threats and violence, especially in situations involving drug affected individuals, gang members, or frustrated family members, makes it challenging to assess and prepare for these occurrences. There is a perceived difference in how they view the violence directed at themselves, depending on whether the person is healthy or for example suffering from a mental illness.

Police officers, trained, equipped, and experienced in uncertain situations, might perceive potentially threatening situations differently, causing unsynchronized risk assessments. The police may feel that their resources are not needed while the participants want reinforcement in the form of the police to get closer to the situation/injury site to be able to care for the patient. In some cases, the situation changed rapidly, and police presence would have been beneficial, as in the case stated below.

*“… when I say the words*,* that he was unconscious*,* then I see in my eyes how the eyes turn completely black on the guy. And he is going completely crazy … … I have time to report to the emergency room because I still have them on the radio*,* and I say ‘yes*,* he’s getting aggressive now.”* [06].

#### Being without police support generates frustration

The participants’ perceptions were that violence in society, in general, is increasing, and assignments are, in one way or another, more often violence-related than before. For example, incidents involving violence causing patient injuries or directed against the participants. Colleagues normally share information, such as locations with higher violence risks, which heightens their awareness of potential risks and threats.

If the participants initially assessed alarms as uncertain, they opted to wait for police response. However, in some cases, they chose to enter risky situations without police support, for instance, to save lives. This elevated risk-taking exposed participants to potential threats and violence while attending to the patient. Experiencing higher risks without police support in their job lead to stress and frustration, prompting thoughts among participants of considering quitting their jobs. This frustration was expressed by the participants, for example when they had to choose between doing their job or staying safe.

*“Yes*,* it is absolutely frustrating because you want to do your job. But at the same time*,* I put my own and my colleague’s safety first*,* as we want to return home after our work shift.”* [07].

When police support is either denied, too far away, or occupied with other tasks, participants expressed being compelled to, in some cases, perform police duties themselves by securing the area or waiting. It is described that the emergency call center urged them to enter and assess situations more thoroughly before assigning a police patrol, creating an unsafe feeling as they lack training or equipment for self-defence while working under uncertain conditions.

*“So*,* it felt a bit like this*,* ‘we should not do this*,* but we have to do it anyway because it takes too long before we get someone else in place’.”* [04].

#### Varying outcomes when cooperating with the police

The participants believe the police play a crucial role in resolving on-site tasks, enabling them to focus entirely on patient care. Police can for example contribute to a safe and calm environment by securing the site and deal with threatening and violent behaviour. The necessity to cooperate with the police is significant, sometimes collaborating as frequently as every work shift in certain areas in Sweden. At times, when the police perceived their presence as unnecessary, participants needed to persistently seek assistance to complete tasks. Participants also describe instances where the police display unfavourable attitudes, appearing stressed, disinterested, or downplaying the severity of perceived threatening situations. Most times, the police presence created a sense of security. However, patients occasionally react negatively to police presence, making care more challenging. Police response may provoke aggression and patients may become aggressive solely due to their presence, as described in the following quote.

*“… you often want the police with you*,* but you don’t want to bring them inside because then the patient perceives a threat.”* [8].

### Strategies for a safe care environment

This category encompasses various experiences utilized to develop strategies for creating a more secure care environment, including prioritizing one’s safety, growing into one’s role, and receiving prior training.

#### Mental preparation and prioritizing personal safety

Participants describe the preparation for potentially threatening and violent situations occurring in several phases before the event. Their mental readiness, along with their colleague’s, during the journey to the incident, was seen as crucial for stress management, avoiding unnecessary risks, and utilizing each other’s experiences. By risk assessing the situation, they weighed their safety against the patient’s. If the situation was deemed unsafe, they opted to wait for the police, regardless of the patient’s injuries. Before entering the situation, they emphasize the importance of surveying and understanding their surroundings to identify potential threats and act proactively. Finding a secure escape route is a priority, along with controlling and adapting to the environment or relocating the patient to a quieter place to ensure safety during care.

When a threat emerged suddenly in a complex situation, participants prioritized their safety and always had a plan in case of escalation. One participant recounted a situation where they had to work without police support, which forced them to resort to using violence to ensure the safety of both the patient and themselves. Participants also describe that, at times, they are compelled to prioritize their safety over patient care in dangerous situations.

*“Threat of suicide – yes*,* it is a huge difference if he has a rope and is about to take his own life with it*,* or if he has a firearm. If he has a firearm*,* no*,* then maybe I do not go in to that patient. Then he may well shoot himself before I go in*,* because I do not expose myself to that danger before the police are in place.”* [05].

#### Impact of previous experience on protective traits

Previous exposure to threats and violence increased self-confidence and facilitated practical work, regardless of whether the previous experience was military-related or not. Some participants considered themselves naive in their early years as ambulance clinicians, believing they now possess better skills to handle threatening and violent situations. This contributes to a solution-oriented attitude and the ability to handle different types of people in unpredictable situations. Those with prior threat and violence exposure during combat or military conditions used that experience to create calmness for themselves.

Participants also develop strategies to adapt to situations. A clear focus, systematic work, and determination created better conditions to manage stress. They gained confidence in their knowledge by trusting themselves or relying on their training and previous experiences.

*“My colleague that I had with me*,* he did his first foreign service*,* and he was terrified as soon as we went out on patrol. … And I thought ‘wow*,* that was nothing’*,* because I felt so safe based on the negative experiences I had*,* which I had turned into positive experiences. Now I felt that ‘I have the equipment*,* I have the education*,* and I have competent people around me’.”* [06].

#### Role allocation and effective communication enhance collaboration

Participants highlighted the advantage of a clear leadership role in complex situations, emphasizing that one ambulance clinician often takes on the role of protector, ensuring security for their colleague to care for the patient without disruption. Working together regardless of personal opinions is deemed vital in an EMS setting. Socializing with colleagues, whether inside or outside work, fostered personal connections that endured during assignments, facilitating seamless collaboration among colleagues and with the police.

Maintaining clear communication was described as crucial for mutual support and cooperation toward common goals, especially in situations where there was a risk of threats and violence. The participants stress that their approach to the patient can significantly influence the situation’s development, either calming or agitating it. Creating understanding in the patient about the situation had a calming effect, and participants have had positive experiences with being clear and transparent. In threatening situations, a less emotionally charged approach could be effective, but adaptability to individual and unique circumstances also played a role.

*“But sometimes you just have to put your foot down and say*,* ‘Now you have to shape up. If you want care*,* it is on my terms. Then you cannot keep on behaving like this or have that attitude’. So being clear and straightforward*,* and not provoking a bad situation. By that*,* I mean it’s unnecessary to start yelling at someone who’s already annoyed. Then you must try to maybe meet each other halfway instead.”* [04].

### Impact during and relief after stressful events

Participants handled stress from threats and violence in different ways; some carried the experience home, while others left it at work. They mentioned three prominent forms of support: collegial, social, and employer organised. Although employer organised support was appreciated, it didn’t always foster enough trust among the participants to be a reliable source of secure relief. Talking about the event with a trusted colleague or understanding partner was highly valued.

#### Desire for specific and recurrent training

There was a desire for de-escalation techniques and self-defence training among participants. Those who had been military trained considered it to be better due to repeated exercises compared to the training in their job as ambulance clinicians. Having an exercise once a year as civilian ambulance clinicians was not considered to have the same effect as practicing regularly in the care team or together with other professions. Participants wished for more frequent and targeted training in threatening and violent situations, preferably alongside police and firefighters.

*“… and then we have exercises far too rarely. We have… well*,* is it a big exercise once every two years? Once a year maybe*,* possibly. And then there are a few colleagues from each station. At least from an ambulance point of view*,* it’s like we have very poor resources to be able to practice.”* [05].

#### Significance of collegial and social support

Participants used various support mechanisms to cope with stress. Continuous support from trusted individuals with whom they have a close relationship was appreciated. Sharing experiences in a relaxed and playful manner also provided relief.

*“I experience that the greatest support*,* I have that with my colleague that I go with. There will be some kind of discussion*,* you can discuss an event for quite a long time. … You still work 40 hours a week together*,* so it becomes a recurring discussion*,* but either through jokes or irony or that you’re talking through the event*,* and there I think you get the best support*,* really.”* [01].

#### Need for organized support

There was a desire for improved employer-organized support following threatening and violent situations. Participants noted that support after such incidents sometimes lacked continuity, with many instances going unnoticed by supervisors. Participants showed a positive attitude toward planned defusing conversations after a critical encounter, especially when involving staff from the event, other agencies, and conversation leaders. The perception of the employer’s view on the need for support had improved, yet shortcomings remain. The planning process was considered slow and unreliable, often unavailable when participants required it. Additionally, there was a discrepancy in the employer’s response to different situations; those involved in threats and violence receive less support compared to those involved in, for instance, fatal incidents. Participants also report experiences of uninvolved managers with the “wrong attitude”. Limited time and resources prevented participants from receiving individual conversations, care, or rest.

*“We do not have the support we may feel that we want. It’s not possible to just call in a new colleague and say*,* ‘I’m not feeling well right now*,* you have to replace me’.”* [05].

## Discussion

The results comprise three main categories: Police cooperation challenges, Strategies for a safe care environment, and Impact during and relief after stressful events, which will be discussed below.

Effective cooperation with the police is often necessary as threatening and violent situations can be unpredictable and change rapidly. However, differences in perceptions can lead to unsynchronized collaboration. While police officers are trained to handle unpredictable situations, they may underestimate the need for their assistance, whereas ambulance clinicians may feel a greater urgency for support.

In some cases, participants were compelled to perform police-related tasks like securing the incident scene or diffusing threats. The results emphasize the critical role of the police in securing the site and, if necessary, using coercive measures. The involvement of the police is crucial in preventing violence directed towards ambulance clinicians [21]. Further research is needed to explore how to synchronize collaboration effectively.

Participants expressed that prioritizing personal safety was essential in threatening situations. They were determined to continue working despite adversity and were willing to take risks. They also highlighted the need for more training to handle such situations. Participants with military experience noted a perceived higher level of training in military forces. For example, standardized training material for the Swedish and American Armed Forces combines “good medicine with good tactics” [22]. The course material for the Prehospital Trauma Life Support (PHTLS) course also prioritizes safety of the caregiver [23].

The participants in this study expressed a desire for training in de-escalation, coping and self-defence to better prepare them for facing threats and violence. As early as 1998, it was noted that ambulance clinicians lacked training and education in how to protect themselves in situations involving threats and violence [24]. A survey of 1,778 participants examined what ambulance clinicians believed would reduce violence. The study showed that training and practice in de-escalating techniques, communication and self-defense were expected to yield positive results. The theme of self-defense included not only physical techniques but also the right equipment and the ability to behave appropriately in threatening situations to create as safe an environment as possible [21]. Although earlier studies have highlighted the need for more training and practice to reduce violence, a relatively recent Cochrane review discovered that although training and education might lead to enhanced personal knowledge and foster more positive attitudes, they did not reduce the occurrence of workplace aggression against healthcare workers [25]. In a study on workplace violence against emergency health care workers, participants had mixed views about extending the training. Some did not believe that additional training would be helpful and felt that dealing with violent situations was not a part of their job [26]. Innovative solutions are needed to improve the way police and ambulance services operate in these scenarios to reduce violence and threats against ambulance clinicians.

In this study, participants explained that by relying on and feeling confident in their training and previous knowledge, they did not feel as vulnerable or victimised as they might otherwise have. Rahmani et al. [27] describe that ambulance clinicians believe that a critical factor affecting the occurrence of violence is their own ability to treat patients and relatives in a way that reduces violence. Similarly, nurses at a Swedish trauma unit felt that their approach to treatment and communication was a key factor in the development of potentially violent situations. They described shifting priorities away from the patient or lying to patients and relatives to avoid conflicts [14].

The results indicate that participants feel stressed when faced with unpredictable threats and violence in their daily work and cope with stress in various ways. Working under sustained high-stress levels can contribute to wear and tear and chronic stress unless nurses are given opportunities to rest. In a study on work-related stress, dealing with death, suffering, and workplace violence ranked among the top three worst experiences for nurses [28]. Another study on posttraumatic stress symptomatology in EMS personnel found that 38% of nurses exposed to threats and/or violence re-experienced the events in line with PTSD criteria, with 2.2% diagnosed with PTSD and 16% meeting clinical anxiety and disability criteria [7].

The results highlighted that informal debriefing among colleagues was highly valued. Discussing experiences with someone who was understanding and close was seen as an effective form of relief. A Swedish study on ambulance nurses’ stress management strategies similarly favoured informal debriefing over organized sessions [29].

This is reflected in the category “Impact during and relief after stressful events”, where participants expressed ambivalence regarding support organized by the employer. While the support was appreciated, it was not always considered reliable, as there was a perception that the employer was unable to foster enough trust among employees. This finding aligns with research indicating that organizational factors such as lack of managerial support and poor leadership negatively impact the health and well-being of ambulance clinicians [30]. The work environment is crucial to nurses and forms the foundation for their ability to work with motivation. Exposure to work-related violence and threats has also been shown to increase the risk of depression [31].

We suggest enhancing managers’ social support, with a focus on fostering trust and enhancing feelings of safety. Additionally, we recommend providing ambulance clinicians with more comprehensive training and simulations covering aspects of situational awareness, handling threats and violence, and de-escalation techniques. Even as these aspects are being enhanced, ambulance clinicians must personally assess whether they are willing to work in environments where they will inevitably encounter threats and violence.

### Limitations

Despite efforts to maintain rigor, we acknowledge several key methodological limitations.

The small sample size of 11 participants may restrict the generalizability of findings beyond Swedish prehospital emergency care. Only two out of eleven of the participants were women, which is not representative of the Swedish EMS.

Caution is warranted when extrapolating results to different contexts.

Interviews and reliance on self-reported data might introduce social desirability and recall bias. Moreover, the virtual format of the interviews could have affected the depth and richness of the data collected compared to face-to-face interactions.

The researchers’ prior experiences in prehospital care could influence their analysis. Furthermore, translation from Swedish to English might introduce subtle linguistic nuances.

These methodological limitations should be considered when interpreting and applying the results of this study.

However, methodological rigour was pursued in various ways. A key strength of this study is the validation of results through triangulation. All authors actively participated in discussions regarding analysis and presentation of results. Additionally, detailed descriptions of participants and context, along with transparency about the authors’ backgrounds, ensured reflexivity.

Future research in this area could benefit from larger and more diverse participant samples, as well as employing additional methodological approaches to enhance rigor and trustworthiness. Further quantitative and mixed methods research is necessary to investigate the rates of violence and threats faced by ambulance clinicians. Another interesting area worth exploring further is the similarity in the experiences between participants with and without military experiences.

### Implications for practice and future research

The findings emphasize the need for practical skill development for ambulance clinicians, such as current treatment and self-defence in threatening situations. Enhancing cooperation with the police through regular, specific exercises can optimize care for the sick or injured. The study results could inform the development of educational concepts to enhance ambulance clinicians’ work environments concerning mental health. Organizational support presents significant developmental potential and merits in-depth investigation.

## Conclusion

This study sheds light on the multifaceted challenges faced by ambulance clinicians in dealing with threatening and violent situations. The findings underscore the necessity for targeted training, improved support systems, and enhanced collaboration with the police to ensure the safety and well-being of both patients and EMS personnel. These insights serve as essential groundwork for further research and tailored interventions in emergency medical services.

## Electronic supplementary material

Below is the link to the electronic supplementary material.

Supplementary Material 1

Supplementary Material 2

## Data Availability

The datasets used and/or analysed during the current study are available fromthe corresponding author on reasonable request.
