# Supplementary material for: Behind the scenes: a qualitative study on threats and violence in emergency medical services
Source: BMC Emerg Med. 2024 Sep 26;24:172. doi: 10.1186/s12873-024-01090-y (PMC11426083; doi:10.1186/s12873-024-01090-y)
Supplement: Supplementary file 1 — Supplementary Material 1 [file 12873_2024_1090_MOESM1_ESM.docx]

Interview Guide

**Regarding informed consent**

Your participation in the study is entirely voluntary, and you may withdraw at any time without providing a reason. If you choose to participate in an interview, everything you say will be treated confidentially, meaning your identity will not be revealed during the processing of the interviews or in the reporting of the results. Unauthorized individuals do not have access to the interviews. The interviews will be de-identified to ensure your identity remains confidential.

**Aim**

The aim of this study is to explore ambulance clinicians’ encounters with threats and violence while providing prehospital care.

**Background** **information**

Age:

Gender:

Years in the profession:

Other healthcare-related education beyond Registered Nurse (RN):

**Opening** **questions**

What is it like to work under threats and violence?

How does it feel to enter a situation involving threats and violence?

Can you describe a complex situation where you worked with prehospital emergency care under threats and violence?

**Continuation**

What are your experiences of working under threats and violence?

How did it feel?

What strategies did you employ?

How did the teamwork function?

What did your training and preparation involve?

What happened afterward, in terms of support?

What lessons do you take away?

**Follow-up questions**

**Area 1:**

Could you share a positive experience of collaboration with colleagues, either professionally or interprofessionally?

Describe a situation where you collaborated. How did you handle it?

Could you share a negative experience of collaboration with colleagues, either professionally or interprofessionally?

What went wrong? How was the situation resolved? Could it have been handled differently? How did it feel when things didn't work out?

**Area 2:**

What experiences did you bring into the situation from previous preparations/training?

How do you perceive the impact of any training, courses, or education?

Can you describe if you had any previous experiences with similar challenges that you could draw upon?

**Area 3:**

Can you describe how you experienced your feelings after handling a situation involving threats and violence?

How has the event affected you professionally?

What experiences/lessons do you take away?

Do you feel it has strengthened you or had a detrimental effect on you in facing future challenges?

How have you received support from your social network or workplace following your experience/experiences?

How was the support perceived?

Can you describe differences in support from the organization and social network?

**Area 4:**

What experiences do you have that are common to this type of situation?

**Closing Question**

Is there anything else you would like to add?

General Follow-up Suggestions:

Avoid yes/no questions.

Can you elaborate further?

What do you mean by that?

Can you develop/describe further?
